# Diagnostic accuracy of multiplex real-time PCR for detecting viruses associated with encephalitis: A systematic review and meta-analysis protocol

**DOI:** 10.1371/journal.pone.0318805

**Published:** 2025-03-13

**Authors:** Sharifah Aliah Diyanah Syed Hussin, Xin Wee Chen, Hassanain Al-Talib, Ang-Lim Chua, Ziauddin Azimi, Seok Mui Wang

**Affiliations:** 1 Institute of Medical Molecular Biotechnology (IMMB), Faculty of Medicine, Universiti Teknologi MARA, Sungai Buloh Campus, Selangor, Malaysia; 2 Department of Public Health Medicine, Faculty of Medicine, Universiti Teknologi MARA, Sungai Buloh Campus, Selangor, Malaysia; 3 Department of Medical Microbiology & Parasitology, Faculty of Medicine, Universiti Teknologi MARA, Sungai Buloh Campus, Selangor, Malaysia; 4 Department of Biochemistry, Faculty of Pharmacy, Kabul University, Jamal Mina, Kabul, Afghanistan; 5 Institute of Pathology, Laboratory and Forensic Medicine (I-PPerForM), Universiti Teknologi MARA, Sungai Buloh Campus, Selangor, Malaysia; 6 Non-Destructive Biomedical and Pharmaceutical Research Center, Smart Manufacturing Research Institute (SMRI), Universiti Teknologi MARA, Puncak Alam Campus, Selangor, Malaysia; Children's National Hospital, George Washington University, UNITED STATES OF AMERICA

## Abstract

**Background:**

Encephalitis is the most common infectious disease of the central nervous system and is associated with high morbidity, mortality, and disability. Therefore, rapid and accurate diagnosis is crucial to provide patients with timely and appropriate therapeutic intervention. In this study, a comprehensive systematic review with meta-analysis will be conducted to summarize the available data and evaluate the diagnostic accuracy of multiplex real-time polymerase chain reaction (PCR) in the detection of viral encephalitis.

**Methods:**

We will search PubMed, MEDLINE, EMBASE, Web of Science (WoS), Scopus and Cochrane Library databases for studies evaluating the diagnostic accuracy of multiplex PCR for the diagnosis of encephalitis caused by viruses from January 2014 to December 2024. Observational study designs with full text will be exported and included. Risk of bias will be assessed using the Quality Assessment of Diagnostic Accuracy Studies (QUADAS-2) tool. Analyses will be performed using the “mada” package of R software (R Foundation for Statistical Computing, Vienna, Austria), and the Summary Receiver Operating Characteristic (SROC) will be calculated using the “midas” package of STATA version 15.0 (Stata Corp., College Station, TX, USA). Certainty of evidence will be performed using Grading of Recommendations Assessment, Development and Evaluation (GRADE) software.

**Results:**

The results will provide clinical evidence for the diagnostic accuracy of the multiplex PCR assay for the detection of viruses that cause encephalitis, including sensitivity, specificity, positive predictive value (PPV), negative predictive value (NPV), positive likelihood ratio (LR+), and negative likelihood ratio (LR-). Finally, we intent to submit this systematic review and meta-analysis to a peer-reviewed journal for publication.

**Conclusion:**

This systematic review aims to provide current evidence for multiplex PCR assay for the diagnosis of viruses causing encephalitis. Importantly, this study focuses on the use of multiplex PCR for viral diagnosis and helps clinicians and patients to better understand its role in the diagnosis of CNS diseases.

**Systematic review registration:**

PROSPERO registration number: CRD42023485942

## 1. Introduction

### 1.1 Background

Encephalitis is an inflammation of part or all of the “encephalon”, i.e., the brain parenchyma [1]. In recent years, this disease has been on the rise and has become the focus of public health attention due to its high morbidity and mortality [2,3]. The worldwide incidence of encephalitis is between 1.5 and 7/100,000 inhabitants/year and the mortality rate is 7% [4]. It is usually caused either by an infectious agent, with viral infections being the most common cause, or by an autoimmune process [5,6]. The most common causes of infective encephalitis globally are herpes viruses, arboviruses, enteroviruses and adenoviruses [5,7]. In general, viruses enter the host outside the central nervous system (CNS) and travel hematogenously or retrogradely from the nerve endings to the brain and spinal cord [8]. When a neurotropic virus invades the brain parenchyma, it infects nearby cells such as microglia, astrocytes, and neurons. This leads to the production of pro-inflammatory chemicals and the subsequent infiltration of immune cells, resulting in brain damage [2]. After the viral infection has subsided, the local immune response can remain active and contribute to long-term neurocognitive impairment, neuropsychiatric disorders and degenerative diseases [9].

Symptoms of encephalitis can range from mild clinical signs such as fever, headache, nausea, vomiting, confusion and altered mental status to more severe symptoms such as seizures, weakness, hallucinations and coma [10,11]. Patients may also experience persistent symptoms such as recurrent headaches, behavioral problems, sleep disturbances, tic disorders and motor disabilities [12]. Various symptoms of neurocognitive impairment such as speech, memory, learning disorders, and attention-deficit/hyperactivity disorder (ADHD) have also been reported as a result of viral encephalitis [12,13]. Encephalitis can affect people of all ages, but often occurs in children and the elderly. Immunocompromised patients are of particular concern as the condition can rapidly progress to severe stages. Therefore, the management of viral encephalitis is very important to improve treatment and prevent exacerbation of the disease, especially in high-risk patients. This disease is easily misdiagnosed due to the common symptoms, which delays treatment. Therefore, early and accurate diagnosis is crucial to provide patients with appropriate treatment and thus reduce their overall morbidity and mortality.

### 1.2 Rationale

Cerebrospinal fluid (CSF) analyses that indicate lymphocytic pleocytosis, normal glucose levels and high protein levels are the primary means of verifying primary viral encephalitis [14,15]. Nowadays, the diagnosis of encephalitis is made by various methods, such as electroencephalography (EEG) and magnetic resonance imaging (MRI) of the brain, as well as by the detection of various pathological changes such as hemiparesis, pyramidal signs and seizures [15,16,17]. However, laboratory methods are needed to determine the possible presence of pathogens causing neuropathology, such as polymerase chain reaction (PCR) assays, serology assays, cultures and microscopy [14,18]. Unfortunately, isolation of viruses was said to be laborious and time-consuming, electron microscopy is less sensitive and requires 10^6^ virions per milliliter in the specimen to detect under the microscope, and immuno-based techniques are unreliable due to their close antigenic similarity and the need for paired serum/CSF [6,19,20].

Multiplex PCR refers to a technique that enables the simultaneous amplification and identification of multiple target sequences in a single reaction tube [21]. This is the main reason why this technique is widely used in the diagnosis of viral pandemics or outbreaks [22]. The simultaneous identification of multiple viruses by multiplex PCR has resulted in a cost-saving, non-invasive and rapid diagnosis that typically takes two hours compared to virus isolation, immunoassay approaches and singleplex PCR, which requires multiple reaction tubes for a comprehensive virus diagnosis [23]. The use of a single specimen for the simultaneous detection of different viruses allows the optimal utilisation of the difficult-to-obtain sample, namely CSF. In addition, immediate detection of the virus allows for more efficient and effective clinical management, reducing treatment costs and the duration of antiviral therapy [24].

Despite the rapid detection of different individual viruses simultaneously, multiplex PCR was said to have lower specificity and sensitivity compared to singleplex PCR [25]. However, some researchers also claimed that multiplex PCR has excellent performance with high analytical sensitivity similar to singleplex PCR and high specificity in detecting viruses such as Japanese encephalitis virus, West Nile virus, hepatitis C virus, chikungunya virus, and yellow fever virus [23]. To address this knowledge gap, we will conduct a comprehensive systematic review with meta-analysis aimed at summarizing the available data and evaluating the diagnostic accuracy of multiplex real-time PCR (self-developed panel by researchers and Biofire FilmArray meningitis/encephalitis panel) in the detection of viral encephalitis compared to the reference standard method.

### 1.3 Objectives

#### 1.3.1 Research questions.

Primary: When compare with reference standard methods, how is the diagnostic accuracy of multiplex real-time PCR in detecting the viruses related to human encephalitis disease?

Secondary:

i. What is the diagnostic accuracy of the multiplex real-time PCR after analysis with adjudication test for discordant results?
ii. Which multiplex panels (Biofire FilmArray meningitis/encephalitis panel vs self-developed panel) showed the highest diagnostic accuracy in detecting viral encephalitis?

#### 1.3.2 Research objectives.

Primary: To determine the diagnostic accuracy of multiplex real-time PCR in detecting the viruses causing human encephalitis.

Secondary:

i. To evaluate the diagnostic performance of multiplex real-time PCR, in case of disagreement between the reference standard method and index tests.
ii. To determine the multiplex panel that showed the highest diagnostic accuracy performance in detecting viral encephalitis.

## 2. Methods

### 2.1 Design and registration

We will perform a systematic review and meta-analysis to evaluate the diagnostic accuracy of multiplex PCR for the detection of encephalitis viruses. This protocol has been registered in the International Prospective Registry of Systematic Reviews (PROSPERO) under the registration number CRD42023485942. In reporting our systematic review, the PRISMA-P (Preferred Reporting Items for Systematic review and Meta-Analysis Protocols) 2015 checklist: recommended items to address in a systematic review protocol is followed (S1 File) [26]. Ethical approval is not required to conduct this meta-analysis.

### 2.2 Information sources

The PubMed, EMBASE, MEDLINE, Web of Science (WoS), Scopus and Cochrane Library databases will be searched for studies evaluating the diagnostic accuracy of multiplex real-time PCR for viral encephalitis disease from January 2014 to December 2024.

### 2.3 Search strategy

The search strategies will be conducted by SAD and WSM using the following keywords, and a similar search will be used for all databases.

#1. “Encephalitis” OR “Central Nervous System disease” OR “cephalitis, phrenitis” OR “viral encephalitis” OR “viral-enceph” OR “viral brain infection” OR “infectious encephalitis” OR “viruses causing encephalitis”.
#2. “Nucleic Acid Amplification Techniques” OR “Multiplex Polymerase Chain Reaction” OR “Multiplex Real-Time Polymerase Chain Reaction” OR “Multiplex Reverse Transcription Polymerase Chain Reaction”
#3. “Diagnostic accuracy” OR “diagnostic performance” OR “sensitivity” OR “specificity” OR “overall accuracy” OR “positive likelihood ratio” OR “negative likelihood ratio”
#4. #1 AND #2 AND #3

### 2.4 Eligibility criteria

#### 2.4.1 Type of study.

Original articles publishing observational study designs (such as cohort studies, case-control and cross-sectional studies) that evaluated the diagnostic performance of multiplex real-time PCR for viral encephalitis will be included. Only freely accessed full text articles subscribed by the institution (Universiti Teknologi MARA, Malaysia) will be included in the review. The studies had to use the index test (multiplex PCR assay) and the reference test simultaneously, which are conventional standard microbiological methods such as CSF/virus cultures, serological methods, singleplex PCR or laboratory-developed tests for viruses. The reference standard should be clearly defined in the study. The articles must provide the data, such as true positive (TP), true negative (TN), false positive (FP), and false negative (FN) values or contain the necessary data to calculate these values. Articles that do not contain raw data or diagnostic test accuracy (DTA) measurements will not be considered unless diagnostic performance results such as sensitivity, specificity, positive predictive value (PPV) and negative predictive value (NPV) have been provided. Exclusion criteria are articles in languages other than English, studies with a sample size of less than ten, conference abstracts, case reports, studies without a reference test and studies with abstracts but without full text.

#### 2.4.2 Participant/population (patients).

Inclusion: All elderly, adults, youngster, children, neonates, and toddler with suspected viral encephalitis. No restrictions regarding gender, age, ethnicity, and nation.

Exclusion: Animals with encephalitis, people with immunodeficiency diseases such as HIV, malnutrition and cancers, or those in an immunosuppressive state due to treatment or medication, who have a brain-related disease that increases the risk of viral encephalitis, people with confirmed bacterial encephalitis, or with confirmed fungal encephalitis.

#### 2.4.3 Index test(s).

Diagnosis by a method that uses a multiplex PCR assay as a diagnostic tool, including the developed multiplex panel (Biofire FilmArray Meningitis/Encephalitis Panel) [27] for the detection of viral encephalitis.

#### 2.4.4 Target condition(s).

Encephalitis is the target condition for this review, and defined as the presence of encephalopathy (a prolonged state of altered consciousness for more than 24 hours, including fatigue, irritability, or changes in behavior or personality) and manifest the proof of CNS inflammation by at least two of:

i. Fever
ii. Seizures or focal neurological findings related to the brain parenchyma
iii. CSF pleocytosis ( > 4 white cells per μL)
iv. EEG results indicate of encephalitis
v. Brain imaging findings showing of encephalitis (swellings).

#### 2.4.5 Reference standards.

The diagnosis of encephalitis depends crucially on lumbar puncture and CSF examination [16]. Laboratory diagnosis is essential to identify the causative agents of CNS infections. It consists of four main methods: (1) isolation of viruses by cell culture technique (gold standard), (2) visualisation of viral morphology under the electron microscope, (3) detection of antiviral antibodies or viral antigens by serological methods, and (4) detection of viral nucleic acids by molecular technique [28].

To assess the diagnostic accuracy of multiplex PCR, several parameters will be used as follows:

(i) True positive (TP) means that both the multiplex PCR assay and the reference standard used detect the exact viral species.
(ii) True negative (TN) will be calculated when both methods are negative/absent for each virus.
(iii) False positive (FP) means that the assay falsely detected the virus as present while the reference method detected it as negative.
(iv) False negative (FN) means that the assay falsely detects the virus as absent while the reference method detects it as present.

Additional reference tests will be included in case of disagreement between reference and index tests, such as additional laboratory tests, clinical analysis and presentation, or CSF cytochemical. For example, a positive clinical diagnosis and a positive reference is considered encephalitis. If both are negative, the disease is categorised as non-encephalitis. In the case of discordant results between index and reference tests, additional reference tests will act as final diagnosis adjudication to confirm the presence/absence of viral detection and further verify the diagnostic accuracy of the index test. This study will tabulate two groups for synthesis: (1) studies that analyse data between the index test and the reference test, and (2) studies that require an adjudication test due to the disagreement between index and reference test. Below are the definitions of the results in the event that the adjudication test is included:

TP: Index test (+) and Reference test (+) and Adjudication test (+)
TN: Index test (-) and Reference test (-) and Adjudication test (-)
FP: Index test (+) and Reference test (-) and Adjudication test (-)
FN: Index test (-) and Reference test (+) and Adjudication test (+)

#### 2.4.6 Study selection.

Primary search results matching the search strategy will be imported into the Endnote Software Version 20 and undergo a deduplication process to eliminate similar article entries. Two investigators (SAD and WSM) will independently screen the eligible studies to ensure transparency and avoid influencing the decision-making process. Screening is two-stages which will begin with a review of titles and abstracts, followed by the full text to confirm the eligibility of studies. Articles that do not fulfil the inclusion criteria will be removed. The reason for exclusion of studies reviewed in full text will be documented. Disagreements between the two researchers will be resolved by discussion with a third researcher (CXW). The eligible articles will be organized and stored in a structured template for data extraction using Microsoft Excel spreadsheet software. With the full-text review, all shortlisted studies will be subjected to secondary validation, which confirms the eligibility of the studies and ensures a solid and reliable database for subsequent analysis and interpretation.

#### 2.4.7 Data extraction.

Two researchers (SAD and WSM) will carry out the data extraction independently of each other. The data will be duplicated and using a pilot extraction form in a Microsoft Excel spreadsheet, the researchers will extract the data consisting of study details such as name of first author, study design, year of publication and country of study origin. Next, the reference standard used, the diagnosis adjudication tests (clinical analysis, CSF cytochemical or additional laboratory test), the viral species detected by each method, the value of TP, TN, FP, and FN of the test, the value of sensitivity (proportion of laboratory test being positive), specificity (proportion of laboratory test being negative), PPV (probability of positive test results is true positive), NPV (probability of negative test result is true negative), inclusion criteria such as the mean age of the participants, and the number of patients/sample size will be extracted. The same researchers will cross-check their respective information from each study. Finally, a third researcher (CXW) will be consulted to resolve the disagreements between the two researchers.

#### 2.4.8 Study duration.

The duration of the study will be two years, and data collection will begin in January 2025.

#### 2.4.9 Risk of bias assessment.

The assessment of the risk of bias (RoB) will be carried out using the Quality Assessment of Diagnostic Accuracy Studies (QUADAS-2) [29]. The two researchers (SAD and WSM) will independently assess the RoB of the relevant literature using a revised QUADAS-2 tool, and disagreements between the researchers will be resolved by discussion with a third researcher (CXW). Assessments will be conducted in four domains of bias: (i) Patient selection, (ii) index test, (iii) different reference standards used, (iv) procedure and timing. Each diagnostic study will be assessed for risk of bias and overall applicability in all four domains. Studies with low risk of bias or concern are categorised as low, while studies with high risk of bias will be categorised as high. Studies may be categorised as unclear if the data are insufficient for analysis and interpretation.

#### 2.4.10 Data synthesis and statistical analysis.

The DTA values of all viruses will be analysed separately for both the reference standard and the adjudication test (if available). We will isolate studies that did not perform the adjudication test due to no disagreement between the index test and the reference test, and studies that require an adjudication test to further assess the index test, in another meta-analysis table. The raw data such as TF, TN, FP, and FN are required to perform the statistical analysis calculations (sensitivity, specificity, PPV, NPV, positive likelihood ratios (LR+) and negative likelihood ratios (LR-)). For the meta-analysis, we will use the bivariate random-effects model to estimate the pooled sensitivity and specificity with the corresponding 95% confidence intervals (95% CI) [30]. We will then generate forest plots with 95% CI for sensitivity and specificity for each study, while the study-specific LR + and LR- will be presented in scatter plots with 95% CI [31]. Summarised sensitivity and specificity will be estimated in Summary Receiver Operating Characteristic (SROC) diagrams [32]. We will calculate the likelihood ratios from the combined sensitivities and specificities. The formula for calculating the LR + is to divide the combined sensitivity by 1- combined specificity. One can calculate LR- by dividing the 1- combined sensitivity by the combined specificity. Heterogeneity between studies will be assessed using the *I*^*2*^ statistic [33]. Significant heterogeneity is indicated by an *I*^*2*^ value of more than 50%, while no heterogeneity is indicated by an *I*^*2*^ value of up to 0%. The availability of more than 10 studies will allow the meta-regression and sensitivity analyses to be performed in case of significant heterogeneity. We will perform subgroup or meta-regression analyses for study year, study site, and study setting (e.g., primary vs secondary vs tertiary care; ward vs intensive care unit (ICU); immunocompetent vs immunocompromised populations; infant vs elderly; multiplex panel (Biofire FilmArray meningitis/encephalitis panel) vs self-developed panel (by researchers). For the sensitivity analysis, we will analyse the articles with a high risk of bias across the four criteria (patient selection, index test, different reference standards used, procedure and timing) to examine their impact on our overall findings. The “mada” pacakage of R software (R Foundation for Statistical Computing, Vienna, Austria) will be used for all analyses, except for the ROC summary, for which the latter will involve the application of the “midas” package in STATA version 15.0 (Stata Corp., College Station, TX, USA) [34]. Finally, assessment of certainty of evidence for the index test will be performed, according to both the reference and adjudication tests using GRADE (Grading of Recommendations Assessment, Development and Evaluation software [35]. The degree of certainty will be assigned as high, moderate, low or very low for the four domains (risk of bias, indirectness, inconsistency/heterogeneity, and imprecision).

## 3. Discussion

Increasing outbreaks of arthropod-borne encephalitis are a growing concern [5]. Encephalitis is a serious disease of the brain that can lead to very serious consequences. An important aspect of the treatment of encephalitis is the management of the long-term brain damage related sequelae, e.g., emotional, behavioural, physical and cognitive impairments. Access to neuropsychological, neuropsychiatric and occupational therapy services, as well as ensuring appropriate aftercare following discharge, are crucial to managing the long-term consequences of the condition. Therefore, early diagnosis prior to treatment is extremely important for prognosis, and the diagnosis of encephalitis is still difficult. Although the etiological agents can be identified by the combination of cytology and biochemical analysis of CSF prior to the conventional methods such as culture and serological assays, these are not independent of being the only diagnostic criteria [36]. Culture of microorganisms is considered the gold standard but is limited by untimely response and meticulous maintenance of viral culture [37]. The multiplex PCR assay is of central importance for the diagnosis of encephalitis, as it can increase the early detection rate. Unfortunately, multiplex PCR also has limitations in the diagnostic field. First, multiplex PCR has less sensitivity than targeted singleplex PCR [38]. This is because of multiple reactions occur in a well, thus can lead to competition for reaction components such as enzymes, cofactor, buffer, and dNTPs [25]. Optimal conditions for each pathogen also may slightly differ, in which primers amplified more efficiently consume more reaction components, which can reduce the yield of other PCR products [39]. Therefore, primer design is critical in the process of developing the multiplex PCR, and it normally require multiple optimizations to find the optimum primer concentration [40]. A study indicates that human parechovirus PCR showed to have higher sensitivity than multiplex PCR [40], concluded that singleplex PCR is required as confirmation test to verify the diagnostic results [41]. But researchers found that multiplex PCR can have similar sensitivity as singleplex PCR by having higher viral load than limit of detection in multiplex PCR assay [42,43].

Second, multiplex panels are designed for qualitative results and cannot provide types or genotype information of the causative pathogens [44]. Unlike multiplex PCR, the sequencing method enables simultaneous analysis of multiple genomic loci and identifies the presence of mutations or locations [45]. Additionally, multiplex PCR is a target-specific technique with limitations in detecting rare or novel viruses [46]. Sequencing is target-independent which able to identify universal pathogens causing CNS diseases within a specimen, simultaneously [47]. For example, metagenomic Next Generation Sequencing (mNGS) has been applied as secondary test for negative result samples obtained using conventional methods [46]. This aims to improve diagnostic outcomes by detecting viruses that may not be detected using target-specific methods, which focus on the most common pathogens [48].

As mentioned in the introduction, this disease is mainly caused by viruses compared to other pathogens such as bacteria, parasites, and fungi. However, most published systematic reviews have not prioritized the assessment of viral diagnosis and, if at all, authors have focused only on specific viruses rather than the common viruses that cause CNS diseases [49]. Therefore, a critical analysis of the performance of multiplex PCR specifically for viral infections leading to encephalitis is important. In addition, many authors prefer to evaluate the FilmArray Meningitis/Encephalitis panel (BioFire Diagnostics®) (FA/ME) alone, without considering the self-developed multiplex PCR as one of the diagnostic tools in their review articles. In one study evaluating the multiplex panel, the certainty of evidence was not assessed, and the findings were not performed simultaneously with both the index and reference tests [50]. Two years later, another improved study was published, which conducted a meta-analysis on specific clinical subgroups and certainty of evidence. Unfortunately, the result was low due to the high risk of bias [27]. Therefore, the added value of our study is that it (i) provides a niche in viral diagnosis with multiplex PCR; (ii) considers both commercial multiplex panel and self-developed panels; (iii) performs a meta-regression analysis on diagnostic performance between multiplex panels (Biofire FilmArray Meningitis/Encephalitis panel) vs self-developed panels; (iv) provides the latest findings on diagnostic accuracy from the current studies. To our knowledge, this is the first diagnostic meta-analysis to focus on the diagnostic efficacy of the multiplex PCR assay, taking into account all of the above criteria. We hope that the outcomes of this study will help clinicians and patients to better understand the role of multiplex PCR in the diagnosis of CNS diseases.

## Supporting information

S1 File PRISMA-P (Preferred Reporting Items for Systematic Review and Meta-Analysis Protocols) 2015 checklist. (DOCX)
